# Effects of Coarse Particulate Matter on Emergency Hospital Admissions for Respiratory Diseases: A Time-Series Analysis in Hong Kong

**DOI:** 10.1289/ehp.1104002

**Published:** 2012-01-20

**Authors:** Hong Qiu, Ignatius Tak-sun Yu, Linwei Tian, Xiaorong Wang, Lap Ah Tse, Wilson Tam, Tze Wai Wong

**Affiliations:** School of Public Health and Primary Care, Chinese University of Hong Kong, Hong Kong Special Administrative Region, China

**Keywords:** coarse particulate matter, emergency hospital admissions, fine particulate matter, generalized additive model, respiratory diseases, time-series study

## Abstract

Background: Many epidemiological studies have linked daily counts of hospital admissions to particulate matter (PM) with an aerodynamic diameter ≤ 10 μm (PM_10_) and ≤ 2.5 μm (PM_2.5_), but relatively few have investigated the relationship of hospital admissions with coarse PM (PM_c_; 2.5–10 μm aerodynamic diameter).

Objectives: We conducted this study to estimate the health effects of PM_c_ on emergency hospital admissions for respiratory diseases in Hong Kong after controlling for PM_2.5_ and gaseous pollutants.

Methods: We conducted a time-series analysis of associations between daily emergency hospital admissions for respiratory diseases in Hong Kong from January 2000 to December 2005 and daily PM_2.5_ and PM_c_ concentrations. We estimated PM_c_ concentrations by subtracting PM_2.5_ from PM_10_ measurements. We used generalized additive models to examine the relationship between PM_c_ (single- and multiday lagged exposures) and hospital admissions adjusted for time trends, weather conditions, influenza outbreaks, PM_2.5_, and gaseous pollutants (nitrogen dioxide, sulfur dioxide, and ozone).

Results: A 10.9-μg/m^3^ (interquartile range) increase in the 4-day moving average concentration of PM_c_ was associated with a 1.94% (95% confidence interval: 1.24%, 2.64%) increase in emergency hospital admissions for respiratory diseases that was attenuated but still significant after controlling for PM_2.5_. Adjusting for gaseous pollutants and altering models assumptions had little influence on PM_c_ effect estimates.

Conclusion: PM_c_ was associated with emergency hospital admissions for respiratory diseases in Hong Kong independent of PM_2.5_ and gaseous pollutants. Further research is needed to evaluate health effects of different components of PM_c_.

Associations between particulate matter (PM) air pollution and cardiorespiratory hospital admissions have been reported by many epidemiological studies over the past two decades (Anderson et al. 2001; Atkinson 2004; Bell et al. 2004; Dominici et al. 2006; Ilabaca et al. 1999; Le Tertre et al. 2002; Lipsett et al. 1997; Morris 2001; Norris et al. 1999; Schwartz and Neas 2000; Slaughter et al. 2005). Most of the studies focused on PM with an aerodynamic diameter ≤ 10 μm (PM_10_) or ≤ 2.5 μm (PM_2.5_). Fewer studies have examined the potential health effects of the coarse fraction (PM_c_; 2.5–10 μm in aerodynamic diameter) and its relationship with cardiorespiratory hospital admissions (Brunekreef and Forsberg 2005; Chen et al. 2005; Halonen et al. 2009; Host et al. 2008; Lin et al. 2005; Peng et al. 2008; Tecer et al. 2008). In addition, excess relative risks (ERRs) estimated for daily respiratory admissions associated with PM_2.5_ and PM_c_ have been quite inconsistent among these studies. A 2005 systematic review of studies on chronic obstructive pulmonary disease (COPD), asthma, and respiratory admissions noted that ERRs in response to short-term exposure to PM_c_ were similar to or larger than corresponding estimates for PM_2.5_ and suggested that PM_c_ might have adverse effects on the respiratory system (Brunekreef and Forsberg 2005). Several studies published after that review also reported significant positive associations between PM_c_ and hospital admissions for respiratory diseases (Chen et al. 2005; Host et al. 2008; Lin et al. 2005; Tecer et al. 2008). On the other hand, the large National Mortality, Morbidity and Air Pollution Study conducted in 108 U.S. urban counties reported a large statistically significant ERR for PM_2.5_ but not for PM_c_ (Peng et al. 2008).

Previous time-series studies on the health effects of air pollution in Hong Kong have focused on PM_10_ because of a lack of PM_2.5_ monitoring data (Ko et al. 2007; Lee et al. 2006; Wong CM et al. 2008a; Wong TW et al. 1999, 2002, 2006). In addition, the Air Quality Objectives (Environmental Protection Department 1987), the national air quality standards for Hong Kong, cover only PM_10_, although the Environmental Protection Department is considering PM_2.5_ regulation as well (Environmental Protection Department 2009). A standard specifically for PM_c_ is not in place or under consideration, but additional studies could help support a PM_c_ standard in the future. In the present study, we conducted a time-series analysis to estimate the health effects of PM_c_ on emergency hospital admissions for total respiratory diseases, COPD, and asthma in Hong Kong after controlling for PM_2.5_ and gaseous pollutants.

## Materials and Methods

**Figure 1:**
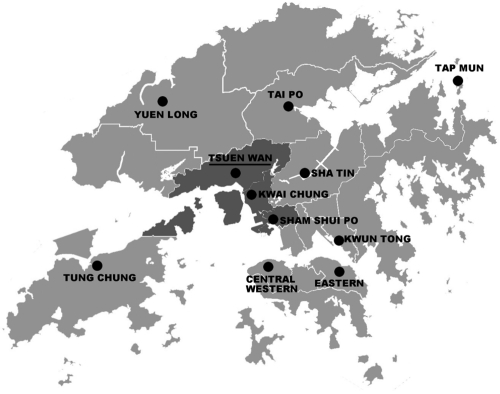
Location of the Tsuen Wan air monitoring station, Tsuen Wan region (dark-gray area), and the other general air monitoring stations (black circles) in Hong Kong.

*Data on particulate pollutants and meteorology variables.* We obtained air pollution data for January 2000 through December 2005 from the Environmental Protection Department. There are a total of 11 general monitoring stations in Hong Kong. All of them monitored PM_10_ and gaseous pollutants [nitrogen dioxide (NO_2_) sulfur dioxide (SO_2_), and ozone (O_3_)] during the study period, but only three (Tsuen Wan, Tap Mun, and Tung Chung) collected simultaneous PM_2.5_ data. The Tap Mun and Tung Chung stations are located in remote areas of Hong Kong, whereas the Tsuen Wan station is located close to the geographic center of Hong Kong (Figure 1) and thus is likely to be more representative of Hong Kong’s air quality in general. In addition, the Tsuen Wan station is not in direct proximity to traffic, industrial sources, buildings, or residential sources of emissions from the burning of coal, waste, or oil. Therefore, instead of estimating average values for the three stations with simultaneous PM_10_ and PM_2.5_ data, we used data from the Tsuen Wan station only. We calculated 24-hr mean concentrations from nonmissing data if at least 18 of 24 hourly concentrations of PM_10_ or PM_2.5_ were available, and we did not impute data for the 195 days with missing PM_c_, which accounted for only 8.9% of the study period. We estimated PM_c_ concentrations by subtracting daily mean PM_2.5_ from PM_10_. In contrast with studies that examined PM_c_ using data collected every 3 or 6 days (Lin et al. 2005; Peng et al. 2008), we analyzed daily PM_c_ data available during the study period. We also calculated daily 24-hr mean concentrations of NO_2_ and SO_2_ and 8-hr mean (1000 hours to 1800 hours) concentrations of O_3_ using data from the Tsuen Wan station and collected daily mean temperature and relative humidity data for the same period from the Hong Kong Observatory.

*Data on hospital admissions.* We collected citywide emergency hospital admissions (admissions through the accident and emergency services) for respiratory diseases in Hong Kong from January 2000 through December 2005. The hospitals included for compilation of hospital admissions were publicly funded hospitals that provide 24-hr accident and emergency services and 90% of hospital beds for Hong Kong residents (Wong et al. 1999). Patient data captured from the computerized medical record system included age, date of admission, source of admission, hospital, residential address, and principal diagnosis on discharge [*International Statistical Classification of Diseases, 9th Revision* (ICD-9); World Health Organization 1975)]. We chose hospital admissions through accident and emergency services for diseases of the respiratory system [ICD-9 codes 460–519, excluding influenza (487.0–487.8)] and for COPD (ICD-9 codes 491, 492, and 496) and asthma (ICD-9 code 493) specifically. We excluded influenza from respiratory diseases because a previous study demonstrated that influenza outbreaks may confound associations between PM and hospital admissions for respiratory diseases (Ren et al. 2006). We also compiled data on emergency hospital admissions for respiratory diseases among patients who are residents of the Tsuen Wan region (TW residents; residential addresses in the area around the Tsuen Wan air monitoring station, including Tsuen Wan, Kwai Tsing, and Sham Shui Po districts; Figure 1), to evaluate the potential influence of exposure misclassification.

*Statistical methods.* We used generalized additive modeling (GAM) with a log link and allowed Poisson autoregression and overdispersion to model the relationship between daily PM_c_ concentrations and health outcomes (Hastie and Tibshirani 1990). All models were adjusted for the day of the week (DOW) and public holidays using categorical indicator variables (Schwartz et al. 1996), and for influenza outbreaks using a dichotomous variable to indicate weeks during which the number of influenza hospital admissions exceeded the 75th percentile for the year (Wong CM et al. 2002). In addition, we used penalized smoothing splines (Host et al. 2008; Kan et al. 2007) to adjust for seasonal patterns and long-term trends in daily morbidity, temperature, and relative humidity with degrees of freedom (df) selected *a priori* based on previous studies (Bell et al. 2008; Peng et al. 2008). Specifically, we used 7 df per year for time trend, 6 df for mean temperature on the current day (Temp_0_) and the moving average for the previous 3 days (Temp_1–3_), and 3 df for humidity (Humidity_0_) on the current day.

The resulting core model to estimate *E*(*Y_t_*), the expected daily emergency respiratory hospital admission count on day *t*, was specified as

log[*E*(*Y_t_*)] = α + *s*(*t*, df = 7/year) + *s*(Temp_0_, df = 6) + *s*(Temp_1–3_, df = 6) + *s*(Humidity_0_, df = 3) + β_1_ × DOW + β_2_ × Holiday + β_3_ × Influenza, [1]

where *s*(·) indicates a smoother based on penalized splines, and β are regression coefficients.

To minimize autocorrelation, which would bias the standard errors, we specified that the absolute values of the partial autocorrelation function for the model residuals had to be < 0.1 for the first 2 lag days (Wong et al. 2008b). When these criteria were not met, we added autoregressive terms for the outcome variable to Equation 1, resulting in the addition of three autoregressive terms (lag_1_, lag_2_, lag_3_) to model emergency hospital admissions for total respiratory diseases, two autoregressive terms (lag_1_ and lag_2_) for COPD, and one autoregressive term (lag_1_) for asthma.

We also estimated the linear effect of PM_c_ according to different lag structures, including single-day lags [current day (lag_0_) up to 5 days before (lag_5_)] and multiday lags (moving averages for the current day and the previous 1, 2, or 3 days: lag_01_, lag_02_, and lag_03_, respectively). However, we focused on 4-day average PM_c_ exposure (lag_03_) for two-pollutant models and sensitivity analyses (Chen et al. 2004). In addition, we estimated the effect of PM_c_ on emergency respiratory hospitalizations after adjusting for exposures to gaseous pollutants (NO_2_, SO_2_, and O_3_). To justify the assumption of linearity between the logarithm of emergency hospital admissions and particle concentrations, we graphically examined concentration–response relationships derived using a smoothing function (Kan et al. 2007; Wong CM et al. 2002).

*Sensitivity analysis.* In addition to analyzing the entire range of particulate concentrations, we estimated effects after excluding days with extremely high or low PM_c_ or PM_2.5_ concentrations (i.e., excluding days with the highest 1% and lowest 1% of values). We also examined the impact of degrees of freedom selection for time trend and weather conditions on PM_c_ effect estimates. To address possible exposure misclassification resulting from the use of pollution data from a single monitoring station, we did a sensitivity analysis restricted to emergency respiratory hospital admissions among TW residents.

We conducted all analyses using the MGCV package in R (version 2.10.0; R Development Core Team, Vienna, Austria). We report results as the percent increase [ERR, with 95% confidence intervals (CIs)] in daily emergency respiratory hospital admissions for an interquartile range (IQR) increase in PM concentrations.

## Results

From 1 January 2000 to 31 December 2005, we recorded a total of 710,247 hospital admissions for respiratory diseases in the study population. Of these, we included 518,864 hospital admissions through accident and emergency services (emergency hospital admissions) in our analyses. On average, there were 237 emergency hospital admissions per day for total respiratory diseases, 81 for COPD, and 20 for asthma (Table 1). The average number of daily emergency respiratory hospital admissions among TW residents was about 50 per day.

**Table 1:** Summary statistics of daily emergency hospital admissions, air pollution concentrations, and weather conditions in Hong Kong, 2000–2005.

No. of days	Percentile				
	Variable	Mean ± SD	Minimum	25th	50th	75th	Maximum
Daily emergency hospital admissions										
Total respiratory diseases		2,192		236.7 ± 55.4		89	198	230	269	518
COPD		2,192		81.1 ± 20.3		22	68	80	95	165
Asthma		2,192		19.6 ± 8.0		1	14	19	25	61
Respiratory diseases in TW residents		2,192		50.0 ± 12.4		18	41	49	57	104
Pollution concentration (μg/m^3^)										
PM_10_		1,998		56.1 ± 27.8		13.5	34.9	49.2	72.5	231.5
PM_2.5_		1,997		39.4 ± 20.7		8.9	23.8	34.8	50.1	179.8
PM_c_		1,997		16.6 ± 9.2		0.8	10.0	14.5	20.9	82.9
NO_2_		1,995		64.4 ± 22.4		13.0	48.4	61.6	77.4	193.9
SO_2_		1,998		22.9 ± 17.1		1.0	11.3	18.3	28.7	143.3
O_3_		1,995		31.1 ± 24.3		1.0	13.2	24.2	42.8	171.7
Meteorology measures										
Temperature (°C)		2,192		23.5 ± 5.0		8.2	19.6	24.9	27.8	31.8
Relative humidity (%)		2,192		78.2 ± 9.7		32	73	79	85	97
Minimum is the lowest value, and maximum is the highest value in the full range.										

Daily mean concentrations of PM_2.5_ and PM_c_ were 39.4 and 16.6 μg/m^3^, with IQRs of 26.3 and 10.9 μg/m^3^, respectively (Table 1). PM_2.5_ accounted for a substantial part of the mass concentration of PM_10_ in Hong Kong: the ratio of PM_2.5_ to PM_10_ ranged from 40% to 98%, with an average of 70%. Therefore, PM_c_ accounted for about 30% of PM_10_ mass concentration. Daily mean concentrations of NO_2_, SO_2_, and O_3_ were 64.4, 22.9, and 31.1 μg/m^3^, respectively (Table 1). PM_10_ was strongly correlated with PM_2.5_ (correlation coefficient, *r* = 0.97) and with PM_c_ (*r* = 0.84), and PM_2.5_ and PM_c_ were moderately correlated (*r* = 0.68; Table 2). Correlation coefficients for PM_c_ and gaseous pollutants were low to moderate (*r* = 0.56 for NO_2_, *r* = 0.27 for SO_2_, *r* = 0.37 for O_3_).

**Table 2:** Pearson correlation coefficients between PM concentrations, gaseous pollutants, and weather conditions.*^a^*

Pollutants	PM_10_	PM_2.5_	PM_c_	NO_2_	SO_2_	O_3_	Temperature
PM_10_	1.000						
PM_2.5_	0.969	1.000					
PM_c_	0.836	0.675	1.000				
NO_2_	0.771	0.786	0.560	1.000			
SO_2_	0.432	0.461	0.267	0.493	1.000		
O_3_	0.475	0.472	0.370	0.303	0.022	1.000	
Temperature	–0.304	–0.285	–0.278	–0.298	0.163	0.054	1.000
Relative humidity	–0.470	–0.409	–0.498	–0.282	–0.062	–0.582	0.213
aAll correlation coefficients except that between O_3_ and SO_2_ are statistically significant (*p* < 0.05).							

**Figure 2:**
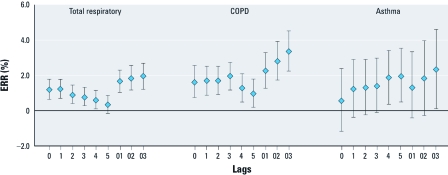
Percent increase (ERR with 95% CI) in emergency hospital admissions due to total respiratory diseases, COPD, and asthma for an IQR (10.9 μg/m^3^) increase in PM_c_ concentrations with different lag days [single lags for the current day (lag_0_) to 5 days before the current day (lag_5_) and multiday lags for the current day plus 1 day before (lag_01_), 2 days before (lag_02_), or 3 days before (lag_03_)].

*Regression results.* PM_c_ was significantly associated (*p* < 0.05) with total respiratory and COPD emergency hospital admissions at most of the lags examined in single-pollutant models, whereas associations with asthma hospitalization were positive but only statistically significant at lag_4_, lag_5_, and lag_03_ (Figure 2). An IQR increase in the 4-day moving average concentration of PM_c_ (lag_03_) was associated with 1.94% (95% CI: 1.24%, 2.64%), 3.37% (2.26%, 4.49%), and 2.32% (0.14%, 4.55%) increases in emergency hospital admissions for total respiratory diseases, COPD, and asthma, respectively (Table 3). After adjusting for PM_2.5_ in two-pollutant models, estimated effects of PM_c_ on respiratory and COPD hospital admissions were attenuated but remained statistically significant, with ERRs of 1.05% (95% CI: 0.19%, 1.91%) and 1.78% (0.41%, 3.16%), respectively. However, the effect estimate for PM_c_ on asthma hospitalizations was close to the null after adjustment for PM_2.5_ (Table 3). Adjustment for gaseous pollutants had little influence on effect estimates for associations between PM_c_ and total respiratory hospitalizations (Table 4).

**Table 3:** Estimated percent increase [ERR (95% CI)] in emergency hospital admissions associated with an IQR increase in PM concentrations, by disease.*^a^*

Pollutant	Total respiratory	COPD	Asthma
Single-pollutant model						
PM_c_		1.94 (1.24, 2.64)		3.37 (2.26, 4.49)		2.32 (0.14, 4.55)
PM_2.5_		2.58 (1.73, 3.44)		4.44 (3.11, 5.80)		4.35 (1.66, 7.11)
Two-pollutant model						
PM_c_		1.05 (0.19, 1.91)		1.78 (0.41, 3.16)		0.27 (–2.42, 3.03)
PM_2.5_		1.81 (0.76, 2.87)		3.13 (1.48, 4.81)		4.14 (0.77, 7.63)
aThe effects of 4-day moving averages (current day to previous 3 days, lag_03_) of daily average PM concentrations were estimated in GAMs, adjusting for time trend, weather conditions, day of week, public holidays, and influenza outbreaks. IQRs: PM_c_, 10.9 μg/m^3^; PM_2.5_, 26.3 μg/m^3^.						

**Table 4:** Adjusted estimated percent increase [ERR (95% CI)] of emergency respiratory hospital admissions associated with an IQR increase in PM concentrations.*^a^*

1st–99th percentile PM_c_ only*c*	In TW residents only*d*	Additionally adjusted for pollutant*b*				
		Pollutant	NO_2_	SO_2_	O_3_
Single-pollutant model										
PM_c_		2.37 (1.51, 3.24)		2.66 (1.33, 4.02)		1.58 (0.86, 2.30)		1.96 (1.26, 2.67)		1.85 (1.15, 2.56)
PM_2.5_		2.55 (1.67, 3.43)		3.02 (1.42, 4.65)		1.98 (1.04, 2.94)		2.74 (1.87, 3.63)		2.43 (1.55, 3.32)
Two-pollutant model										
PM_c_		1.32 (0.23, 2.42)		1.78 (0.11, 3.47)		1.07 (0.21, 1.94)		1.02 (0.16, 1.89)		1.08 (0.22, 1.95)
PM_2.5_		1.70 (0.59, 2.82)		1.72 (–0.26, 3.74)		1.19 (0.05, 2.33)		1.97 (0.89, 3.06)		1.62 (0.53, 2.71)
aThe effects of 4-day moving averages (current day to previous 3 days, lag_03_) of daily average PM concentrations were estimated in GAMs, adjusting for time trend, weather conditions, day of week, public holidays, and influenza outbreaks. IQRs: PM_c_, 10.9 μg/m^3^; PM_2.5_, 26.3 μg/m^3^. bAnalysis covered the entire range of PM_c_ concentration and citywide respiratory admissions. cAnalysis restricted to 1st–99th percentiles (6.42–42.96 μg/m^3^) of PM_c_ concentration. dAnalysis restricted to hospital admissions in TW residents.										

**Figure 3:**
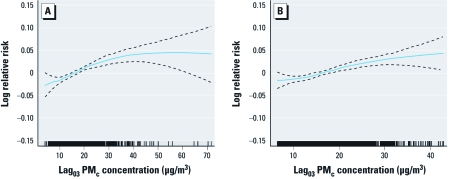
Concentration–response curves between the logarithm of emergency respiratory hospital admission and PM concentration (df = 3). The density of the vertical bars on the x-axis shows the distribution of pollutant concentration. GAMs were used, adjusting for time trend, weather conditions, day of week, public holidays, and influenza outbreaks. (*A*) Analysis covering the entire range of PM_c_ concentrations. (*B*) Restricted analysis excluding days with the lowest 1% and the highest 1% PM_c_ concentrations.

The concentration–response curve for PM_c_ and emergency hospital admissions for total respiratory diseases tended to plateau at higher concentrations of PM_c_, but estimates were imprecise because of limited data in this range (Figure 3A). After we excluded the highest 1% and the lowest 1% extremes of PM_c_ concentrations, the curve appeared essentially linear (Figure 3B). The estimated effect (slope) of PM_c_ modeled as a linear variable increased slightly after excluding days with extreme concentrations, both before and after adjustment for PM_2.5_ (Table 4).

**Figure 4:**
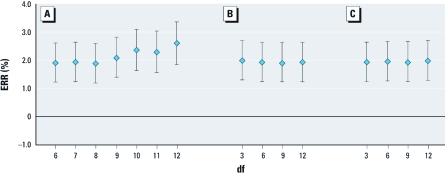
Sensitivity analyses for varying degrees of freedom for time trend and weather conditions on emergency respiratory hospital admissions based on an IQR increase of lag_03_ PM_c_ concentrations: df = 6–12 per year for time trend (*A*), df = 3–12 for current day and previous 3 days’ mean temperature (*B*), and df = 3–12 for current day relative humidity (*C*).

Varying the degrees of freedom for time trend (within the range of 6–12 per year) and weather conditions (mean temperature and humidity, within the range of 3–12) did not affect the regression results substantially (Figure 4), suggesting that effect estimates for PM_c_ were relatively robust to changes in degrees of freedom for model covariates. ERR estimates based on data restricted to emergency respiratory hospitalizations among TW residents were less precise but slightly higher than corresponding estimates based on all observations.

## Discussion

This study is one of the few to investigate the association between PM_c_ and respiratory hospitalizations. We found significant positive associations between PM_c_ concentrations and emergency hospital admissions for respiratory diseases in Hong Kong. To our knowledge, this study is the largest single-city study to date of the effects of PM_c_ on emergency hospital admissions for respiratory diseases, including more than half a million admissions over 6 years. In contrast with studies based on PM data collected every third or sixth day (Lin et al. 2005; Peng et al. 2008), we evaluated daily data and were able to estimate effects of multiday average concentrations of PM_c_, which were larger in magnitude than estimated effects of single-day lags in most cases. We estimated statistically significant positive associations between PM_c_ and emergency hospital admissions for total respiratory diseases and COPD for almost all lags examined. The estimated effect of PM_c_ on asthma appeared to be strongest several days after exposure, consistent with a previous study (Lin et al. 2002).

Positive associations between PM_c_ and total emergency respiratory hospitalizations, especially COPD, remained after adjusting for PM_2.5_, but the estimated effect of PM_c_ on emergency asthma hospitalizations was close to the null after adjusting for PM_2.5_. A few studies estimated effects of PM_c_ on respiratory admissions after adjusting for PM_2.5_ (Burnett et al. 1999; Chen et al. 2004; Ito 2003; Peng et al. 2008), but only one (Burnett et al. 1999) reported statistically significant associations independent of PM_2.5_. However, unlike the daily measurements used in our study, daily levels of PM_c_ and PM_2.5_ in that study were estimated from 6-day sampling and not directly measured. Two studies have reported positive associations between PM_c_ and asthma hospitalization in children, but estimates were not adjusted for PM_2.5_ (Lin et al. 2002; Tecer et al. 2008). Estimated effects of PM_c_ changed very little after we adjusted for possible confounding effects of gaseous pollutants (NO_2_, SO_2_, O_3_), and others have also reported positive associations between PM_c_ and respiratory hospitalizations after adjusting for gaseous pollutants (Chen et al. 2004, 2005; Lin et al. 2002, 2005). The correlation coefficients between PM_c_ and gases in these Canadian studies were low to moderate, consistent with our study (correlation coefficients ranging from 0.27 for PM_c_ and SO_2_ to 0.56 for PM_c_ and NO_2_).

Englert (2004) suggested that the relative sizes of effects attributed to fractions of PM_10_ depend on their relative mass percentages. Although PM_c_ represented only about 30% of the PM_10_ mass concentration in our study, we estimated statistically significant ERRs for emergency respiratory hospital admissions in association with PM_c_, which supports a specific effect of this PM fraction.

The concentration–response relationship between PM_c_ and emergency hospital admissions for total respiratory diseases was almost linear after excluding the highest 1% and the lowest 1% extreme concentrations of PM_c_, and the slope of the estimated association based on a linear model increased slightly. Our results were not substantially modified when we varied the degrees of freedom for smoothers of time and weather conditions. Analyses restricted to emergency hospitalizations among residents living near the monitoring station also were consistent with the overall results, which supports the use of PM data from a single central monitoring station in our main analyses.

Effects may vary for PM_c_ from different sources and with different chemical compositions, and it has been proposed that differences in associations estimated for Hong Kong and U.S. populations (Peng et al. 2008) might be explained by differences in PM_c_ composition. Further studies are needed to examine the health effects of the specific components in PM_c_.

Smaller particles offer a proportionally larger surface area resulting in potentially higher concentrations of adsorbed or condensed toxic air pollutants per unit mass. Hence, PM_2.5_ is frequently assumed to be a more relevant exposure indicator than are larger particles. However, the pathological mechanisms of particles on human health are not fully understood. Particle size may be associated with chemical, biological, and physical properties that contribute to specific pathological mechanisms. PM_c_ originates mainly from the soil and abrasive mechanical processes and thus may carry biological materials such as bacteria, molds, or pollens that can produce adverse health effects in the respiratory system (Almeida et al. 2006). Our results lend support to possible adverse health effects of PM_c_ exposure that are independent of PM_2.5_ and gaseous pollutants. Further study of seasonal differences in PM_c_ composition and season-specific PM_c_ effects may help clarify pathological mechanisms.

Some limitations of the present study should be noted. We estimated PM_c_ concentrations by subtracting PM_2.5_ from PM_10_ measurements. A disadvantage of this method is that PM_c_ exposure estimates are subjected to two sources of random error in measurement (standard error) rather than one, which may reduce the statistical power of detecting an association. Because we still observed significant associations between PM_c_ and emergency respiratory hospital admissions in Hong Kong, these were likely true associations. As in other time-series studies, we used available outdoor monitoring data to represent the population exposure to ambient particles. Indoor air pollution and personal exposure data were not available. A simulation using data from a recent multipollutant (PM_2.5_, O_3_, and NO_2_) exposure assessment study conducted in Baltimore, Maryland (USA), suggested that for PM_2.5_, ambient concentrations available from local monitoring stations might be adequate surrogates for total personal exposures (Schwartz et al. 2007). On the other hand, PM_c_ levels tend to be less spatially homogeneous than PM_2.5_ (Monn 2001), increasing the likelihood that personal exposure will be misclassified in monitor-based studies of ambient PM_c_.

In conclusion, we found evidence indicating that PM_c_ may play an important role in emergency hospitalizations for respiratory diseases independent of PM_2.5_ and other gaseous pollutants. Our findings in Hong Kong add to the growing body of literature concerning adverse health effects of PM_c_. However, further studies are needed to elucidate toxicological differences related to effects of PM_c_ with different compositions under different situations of time and place and to identify PM_c_ component(s) posing the greatest health risk.
